# Novel small molecules disrupting polarized cell expansion and development in the moss, *Physcomitrium patens*

**DOI:** 10.5511/plantbiotechnology.25.0209a

**Published:** 2025-06-25

**Authors:** Prerna Singh, Naoya Kadofusa, Ayato Sato, Satoshi Naramoto, Tomomichi Fujita

**Affiliations:** 1 Graduate School of Life Science, Hokkaido University; 2 Institute of Transformative Bio-Molecules (WPI-ITbM), Nagoya University; 3 Center for One Medicine Innovative Translational Research (COMIT), Nagoya University; 4 Faculty of Science, Hokkaido University; 5 JST-PRESTO

**Keywords:** cell polarity, chemical screening, *Physcomitrium patens*, tip growth

## Abstract

Tip growth is vital for plant growth and development, yet the regulatory mechanisms governing this process remain incompletely understood. In this study, we identify Reagent F4, a novel small molecule that disrupts tip growth and polarized cell expansion in the moss, *Physcomitrium patens* protonemata. Through unbiased chemical screening, we found that Reagent F4 induces abnormal protonemal morphology, characterized by reduced cell elongation and stunted cell expansion. Our analyses revealed that F4 treatment triggers actin depolymerization and disrupts apical actin foci, which are critical for initiating and maintaining tip growth. Additionally, both acute and prolonged F4 exposure led to mislocalization of ROP GTPase, a key regulator of cell polarity. Transcriptomic analyses of F4 treated protonemata show significant downregulation of genes involved in lipid asymmetry, a process essential for polarized growth. These findings establish Reagent F4 as a valuable tool to investigate the molecular mechanisms governing tip growth in *P. patens* and highlight the potential role of lipid asymmetry in coordinating cytoskeletal organization and membrane polarity.

## Introduction

In multicellular eukaryotes, cell polarity is crucial for various cellular, tissue, and organism-level functions and physiology, including responses to the external environment. These properties are significant for developmental processes, such as establishing the body plan and forming organizational systems (Gorelova et al. 2021; Guo and Dong 2022). The establishment and maintenance of cell polarity represent a highly conserved and intricate mechanism that relies on the precise delivery of intracellular signals and materials. The establishment of cell polarity is essential for conferring specialized functions to various cell types. For example, the polar localization of auxin transporters, such as PIN-FORMED (PIN) proteins, drives the polar movement of auxin within various tissues in *Arabidopsis thaliana* (Michniewicz et al. 2007); the polar localization of BOR1 facilitates the polar transport of boron in the endodermis of roots (Takano et al. 2010). Additionally, transient polar localization of BASL protein facilitates asymmetric cell division of stomatal-lineage cells (Dong et al. 2009). Root hairs, pollen tubes in seed plants, and protonemal filaments in mosses extend through a process called tip growth. This growth requires a polarized intracellular organization that delivers cell membrane and cell wall materials to the apical region, thereby sustaining polarized cell growth (Hepler et al. 2001).

The moss, *Physcomitrium patens*, is an excellent model plant to study mechanisms of tip growth in plants; *P. patens* spends a majority of its gametophytic life stage in the juvenile, protonemal form, where the protonemata display distinct unidirectional and anisotropic growth patterns by undergoing tip growth via polarized cell expansion and asymmetric cell division (Menand et al. 2007; Naramoto et al. 2022; Rensing et al. 2020). Actin is vital for tip growth in bryophytes like *P. patens* (Augustine et al. 2008). Pharmacological inhibition of actin cytoskeleton or mutants defective in the function of actin-binding proteins (Augustine et al. 2008; Vidali et al. 2007) or actin nucleating factors (Vidali et al. 2009) exhibit a loss of polarized growth. Another cytoskeletal component, microtubules, help in establishing the tip growth direction in *P. patens* (Yamada and Goshima 2018), while also intersecting with the polarization of actin clusters in the apical cells, facilitated by myosin VIII and kinesin proteins (Wu and Bezanilla 2018; Yamada and Goshima 2018). Furthermore, polarized growth is also influenced by exocytosis and specific cell wall components along with their modifications (Ye and Zhong 2022). One of the key signaling molecules involved in regulating cell growth and polarization in tip-growing cells of moss protonemata is the CDC42/RHO/RAC-like small GTPases known as RHO-related GTPases of plants (ROPs). In *P. patens* protonema, ROP polarizes in a compact apical gradient in the plasma membrane of tip-growing cells (Cheng et al. 2020; Ito et al. 2014; Yi and Goshima 2020). Loss-of-function mutants of ROPs and their effectors compromise polarized tip growth in *P. patens* (Burkart et al. 2015; Cheng et al. 2020; Yi and Goshima 2020). The polarized distribution of ROP is mediated through interactions with lipids facilitated by the polybasic hypervariable region and a carboxyl-terminal CAAX prenylation motif. While the importance of the prenylation signal in the proper localization of ROP is known (Yi and Goshima 2020), the mechanisms underlying ROP-membrane interaction in *P. patens* are not fully understood.

Over the last two decades, classical genetics has served as the primary means to unravel the mechanisms underlying cell polarity (Dong et al. 2009; Müller et al. 1998; Takano et al. 2010). Although this approach has yielded valuable insights, it has limitations. For instance, plant genomes often exhibit high genetic redundancy, resulting in loss-of-function mutants displaying phenotypes similar to the wild type (Borevitz and Ecker 2004). Additionally, creating a complete collection of loss-of-function mutants for essential genes may lead to lethality. Both redundancy and lethality are frequently encountered in genes that regulate essential cellular functions. Chemical genetics offers a promising alternative to address these challenges (Schreiber 1998). The cellular responses can be finely tuned by applying small molecules in a spatially and temporally controlled manner. In this study, we employed a novel chemical called F4 to explore the mechanisms behind polarized cell expansion in the moss *P. patens*. The reagent F4 disrupts actin organization and leads to a loss of apical localization of ROP small GTPases. The unique structure of this compound suggests that F4 may interact with novel components or pathways related to cell polarity. Our transcriptome analyses indicate that Reagent F4 might be involved in the maintenance of lipid asymmetry via flippase activity. Thus, we propose the novel chemical Reagent F4 to investigate new aspects of lipid asymmetry in coordinating cytoskeletal dynamics and membrane polarity.

## Materials and methods

### Plant materials and growth

The moss *Physcomitrium patens* Bruch & Schimp subsp. *patens* was used as the wild-type (WT) strain in this study (Ashton and Cove 1977; Rensing et al. 2020). LifeAct-Venus, a marker for actin filaments (Era et al. 2009), and GFP-Tubulin, a marker for microtubules (Hiwatashi et al. 2008), were employed to visualize the cytoskeleton. ROP4swmNG was utilized as a marker for ROP GTPases in *P. patens* (Cheng et al. 2020). WT and transgenic marker lines were homogenized in sterile water and plated on BCDAT or BCD with 0.8% (w/v) agar medium. Cultures were maintained under continuous white light (ca. 25 µmol photon m^−2^ s^−1^) at 25°C (Nishiyama et al. 2000). Protonemal tissues from 4- to 5-day-old subcultures were utilized for subsequent experiments.

### Chemical screening

For screening as well as structure-activity relationship (SAR) analysis (McKinney et al. 2000), WT protonemal tissues were inoculated into 96-well plates with glass coverslip bottom (Iwaki), each containing 80 µl of BCDAT medium supplemented with 0.5% (w/v) glucose (BCDATG) and 0.5% (w/v) gellan gum (Wako). The plates were incubated upside down under red light (ca. 27 µmol photon m^−2^ s^−1^) to suppress side branch formation at 25°C for 5 days. Screening for small molecules that induce defects in tip growth and/or cell expansion was conducted using the chemical library provided by ITbM, Nagoya University, which consisted of 2000 compounds dissolved in DMSO at a stock concentration of 10 mM. For the chemical screening experiments, the compounds were diluted in liquid BCDAT medium containing 0.5% (w/v) glucose (BCDATG) to a working concentration of 100 µM. Following the 5-day incubation of WT samples under red light, the diluted small molecules were added to the wells to a final concentration of 10 µM. Samples were then incubated for another 2 days under continuous white light, with 0.1% (v/v) DMSO as the negative control. Observations were conducted every 24 h over the 2-day treatment period using a fluorescence microscope (Olympus BX60). For SAR analysis, Reagent F4 analogs were dissolved in DMSO at a stock concentration of 10 mM, then diluted in liquid BCDATG. The diluted solutions were added to wells to achieve a final concentration of 100 µM, with 100 µM Reagent F4 as a positive control and 0.1% (v/v) DMSO as a negative control. Higher concentrations of compounds were used for SAR analysis to ensure a clear phenotype, compared to those used in chemical library screening.

### Microscopy

To assess the dose-dependent effects of Reagent F4 and conduct the SAR assay, the subapical cell length of protonemal tissues was measured using an Olympus fluorescence microscope (Olympus BX60) with a 10× objective. For live imaging of cytoskeletal elements and ROP localization under the influence of Reagent F4, protonemal cells were cultured in 35-mm Petri dishes with a 27-mm glass coverslip at the bottom (Iwaki). The culture medium consisted of 500 µl of BCD with 0.8% (w/v) agar or BCDATG with 0.5% (w/v) gellan gum (Wako) and samples were grown under continuous white light (for BCD agar cultured samples), and red light (for BCDATG gellan gum cultured samples), for 5–7 days. Reagent F4 and DMSO were diluted in liquid BCD/BCDATG and applied exogenously to final concentrations of 100 µM and 0.1%, respectively. Latrunculin B and Oryzalin were also diluted in liquid BCD and applied exogenously to final concentrations of 1 µM and 10 µM, respectively. Cytoskeletal elements (LifeAct-Venus, GFP-Tubulin), ROP (ROP4swmNG) and septa and cell walls stained with propidium iodide (PI) were visualized using an inverted spinning disk confocal microscope (X-Light V3, CrestOptics) equipped with a 1.40 NA 60× oil immersion objective (Plan Apo VC 60×, Nikon) and 0.60 NA 40× objective (S Plan Fluor ELWD 40×, Nikon). Fluorescent markers were excited using a 470 nm laser and PI was excited using a 555 nm laser, while chlorophyll autofluorescence was excited with a 640 nm laser. Emission filters were set to 485–535 nm for GFP, Venus, mNeonGreen, 560–620 nm for PI and 660–735 nm for chlorophyll autofluorescence. Image acquisition was managed via MetaMorph software (Molecular Devices). Quantification of filament density (amount of cytoskeletal components per unit area in specific cell regions) and skewness (statistical parameter that quantifies fluorescence distribution asymmetry where normal cytoskeletal filaments exhibit a normal distribution of fluorescence intensities and an increase in brighter pixels from bundling shifts, leading to higher skewness values) was done as described by Higaki (2017), and normalized mean intensity graphs of ROP apical gradient were generated by measuring the signal intensity along a manually drawn line tracing the plasma membrane of the apical cell starting from the left lateral side of the cell to the right lateral side of the cell as described by Cheng et al. (2020). All microscopy data analyses were performed using Fiji (ImageJ) software (Schindelin et al. 2012).

### FRAP assay

Apical cells in samples grown in BCDATG (0.5% (w/v) glucose supplemented with 0.5% (w/v) gellan gum) under red light for 5–6 days were observed using a Zeiss LSM 980 laser-scanning confocal microscope with a Plan Apochromat 63×/1.40 oil immersion objective. A circular stimulation ROI with a diameter of 3 µm was placed at the apical plasma membrane where the ROP signal was the strongest. The 488 nm laser at 100% power was used for photobleaching stimulation. Before stimulation, images were taken every 2 s for 10 s, followed by stimulation for 2 s, and the imaging continued after stimulation every 2 s for a total of 1.5 min. The mean fluorescence intensity of the ROP4swmNG signal was measured within the stimulation ROI. For each cell, the mean intensity measurements for the first five time points before stimulation were averaged to generate the reference intensity, and the mean intensity value of every time point in the movie was divided by the reference intensity value to create a normalized mean intensity. Graphs were generated by averaging each cell’s normalized mean intensity and plotting versus time. Photobleaching of the apical cell was started 30 min after exogenous application of the compound and the control.

### RNA sequencing

Total RNA was extracted from protonemal tissues treated with 100 µM Reagent F4 using the RNeasy Plant Mini Kit (Qiagen). Tissues were collected at 1, 3, 12, and 24 h after treatment. Protonemata treated with 0.1% (v/v) DMSO for 24 h served as the control. Three biological replicates were sampled for each treatment and time point. Following RNA quality assessment, sequencing libraries were prepared using the Lazy-Seq protocol (Kamitani et al. 2019). High-throughput RNA sequencing was conducted on the NovaSeqX Plus platform (Illumina) in 150-bp paired-end mode.

### RNA sequencing analysis

After sequencing, raw data were obtained in FASTQ format, and data quality was assessed using FastQC (Andrews 2010) and MultiQC (Ewels et al. 2016). Read trimming was performed with Cutadapt (Martin 2011). Transcript abundances were quantified using Kallisto (Bray et al. 2016) by aligning the reads to the *Physcomitrium patens* genome v3.3, obtained from Phytozome (https://phytozome-next.jgi.doe.gov/ (Accessed Dec 12, 2023)). Transcript abundance estimates were imported and summarized using the *tximport* package (Soneson et al. 2016), followed by differential gene expression analysis conducted with DESeq2 (Love et al. 2014). All analyses were performed on the Galaxy platform (Afgan et al. 2016). Subsequent hierarchical clustering of normalized counts and Gene Ontology (GO) term enrichment analysis were carried out using iDEP 2.0 (Ge et al. 2018) and ShinyGO 0.81(Ge et al. 2020). Hierarchical clustering was done with the Euclidean distance measure with complete linkage and values were plotted as TPM values.

### Statistical analysis

One-way ANOVA with Tukey’s post-hoc test was performed using GraphPad Prism version 10.4.1 for Windows, GraphPad Software, www.graphpad.com. Details about the statistically significant differences are described in the figure legends.

## Results

### Identification of small molecules that affect tip growth in *P. patens*

**Figure 1:**
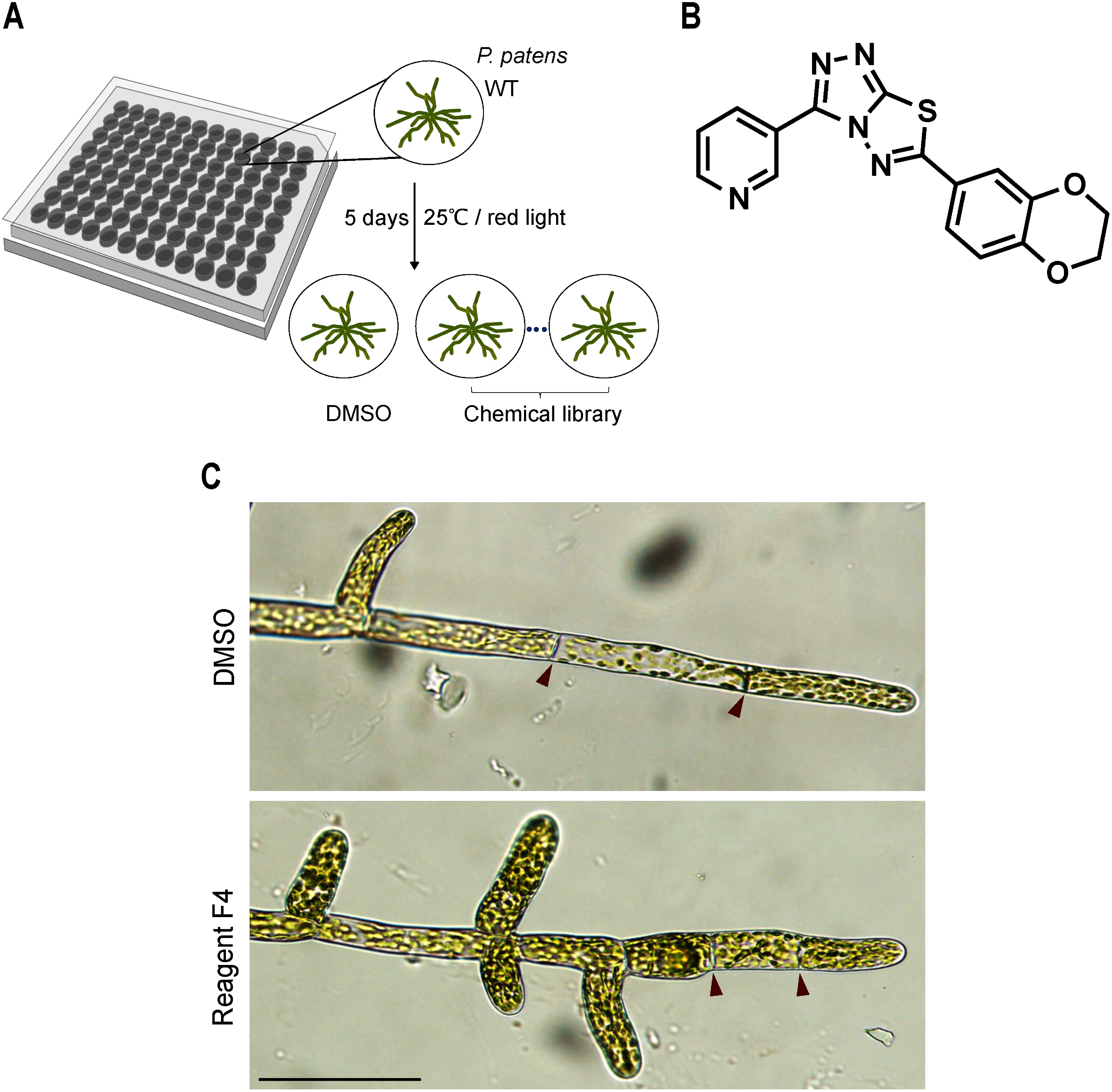
Figure 1. Chemical screening for compounds that affect tip growth. A. Schematic representation of the phenotype-based screen using wild-type (WT) *P. patens* protonemata. Samples were first incubated under red light for five days to suppress side branch formation and then treated with the chemical library, followed by incubation under white light for two days. B. Chemical structure of Reagent F4. C. Brightfield images of WT protonema treated with 0.1% DMSO as a control and 10 µM Reagent F4 after one day of treatment. Red arrowheads indicate septa. Scale bar, 100 µm.

To identify novel factors that affect tip growth and polarized cell expansion in *P. patens* protonemata, we established a chemical screening method utilizing an unbiased chemical library of 2000 compounds. The screening aimed to identify compounds that induce aberrant protonemal morphologies, using dimethyl sulfoxide (DMSO) as a mock control. Wild-type (WT) protonemata were cultured in 96-well plates under red light for 5 days to suppress side branch formation and slow down the growth of moss protonemata to observe better any changes induced by the chemicals. Then, different compounds at a final concentration of 10 µM were added to each well, and finally cultured under white light for two additional days (Figure 1A). This led to the identification of one of the triazolothiadiazole derivatives, 6-(2,3-dihydro-1,4-benzodioxin-6-yl)-3-(3-pyridinyl)-[1,2,4] triazolo[3,4-b] [1,3,4] thiadiazole, referred to as “Reagent F4” (Figure 1B). Application of Reagent F4 resulted in a marked reduction in cell length and swollen cells with stunted cell expansion of protonemata (Figure 1C), compared to the mock samples. With no prior biological activity reported for Reagent F4, our results suggest that it might act as an inhibitor of polarized growth in *P. patens* protonemata, potentially through mechanisms that are novel and yet to be characterized.

### Reagent F4 inhibits cell elongation in *P. patens*

**Figure 2:**
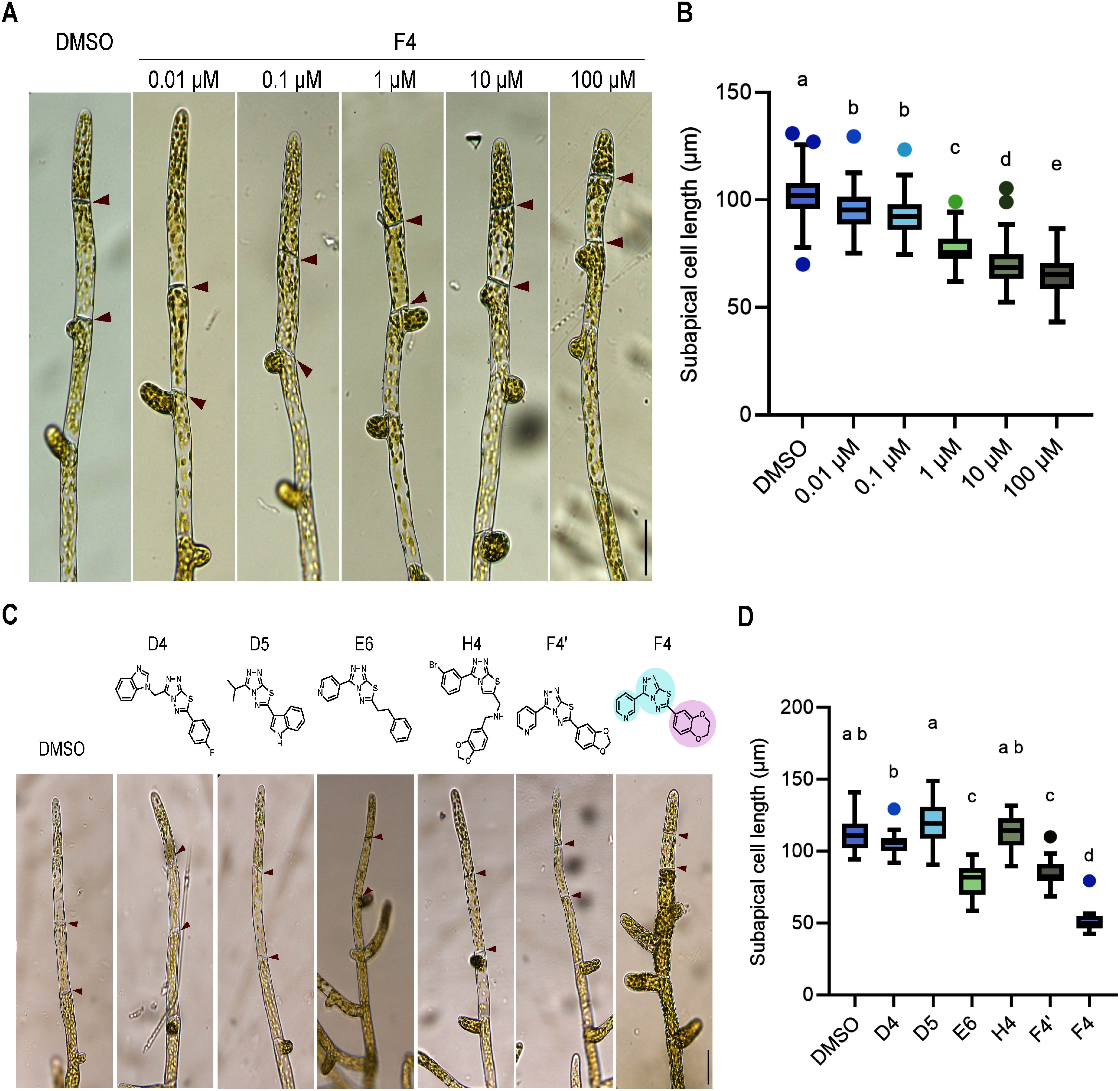
Figure 2. Reagent F4 affects cell elongation in a dose-dependent manner. A. Brightfield images of WT protonema treated with 0.1% DMSO as control and different concentrations of Reagent F4, after one day of treatment. Red arrowheads indicate septa. Scale bar, 50 µm. B. Quantification of subapical cell length after treatment with 0.1% DMSO and different concentrations of Reagent F4, after one day of treatment (*n*=130, 119, 109, 98, 115, 105). Different letters indicate statistically significant differences (one-way ANOVA and Tukey’s multiple comparison test *p*<0.01). C. Chemical structures of Reagent F4 analogs with corresponding brightfield images of WT protonema treated with 100 µM of each compound. Red arrowheads indicate septa. Scale bar, 50 µm. D. Quantification of subapical cell length after treatment with 0.1% DMSO and different analogs of Reagent F4, after two days of treatment (*n*=14, 19, 15, 10, 17, 17, 11). Different letters indicate statistically significant differences (one-way ANOVA and Tukey’s multiple comparison test *p*<0.01).

To further evaluate the effects of Reagent F4, we specifically examined its impact on the morphology of protonemata, focusing on the length of subapical cells. Subapical cells, which are positioned below the apical cells in the protonemal structure, undergo minimal expansion following cell division. Our observations revealed a clear dose-dependent reduction in the length of subapical cells in protonemata treated with Reagent F4. Specifically, as the concentration of Reagent F4 increased, we noted a corresponding decrease in the length of the subapical cells. This effect was observed in both the second cell (1st subapical cell) and the third cell (2nd subapical cell) from the apical cell, strongly indicating that this reagent acts as an inhibitor of cell elongation (Figure 2A, B, Supplementary Figure S1).

In addition to assessing the morphological impacts, we aimed to pinpoint the specific molecular structure responsible for the observed biological activity of Reagent F4. To achieve this, we conducted a structure-activity relationship (SAR) analysis using five analogs of Reagent F4. We aimed to identify F4 analogs that induced phenotypes similar to F4, upon application to *P. patens* protonema. Through this analysis, we identified compounds E6 and F4′ that showed a reduction in the subapical cell length, a phenotype also observed upon Reagent F4 treatment (Figure 2C, D). Since Reagent F4, E6, and F4′ share common moieties consisting of two heteroaromatic rings in their chemical structures, we conclude that these heteroaromatic rings are likely essential for the biological activity of Reagent F4 (marked in blue, Figure 2C). Although compounds E6 and F4′ phenocopy Reagent F4′s effect on *P. patens* protonema, the reduction of subapical cell length is not as strong as that observed in samples treated with F4. The chemical structures of F4, E6, and F4′ differ in their -R group (marked in pink, Figure 2C). The difference in the intensity of the phenotype induced by the compounds and the difference in the -R group suggests that the -R group attached to the core structure might play a role in modulating the intensity of the chemical’s effects (Figure 2D).

### Reagent F4 treatment leads to actin depolymerization and loss of actin foci

**Figure 3:**
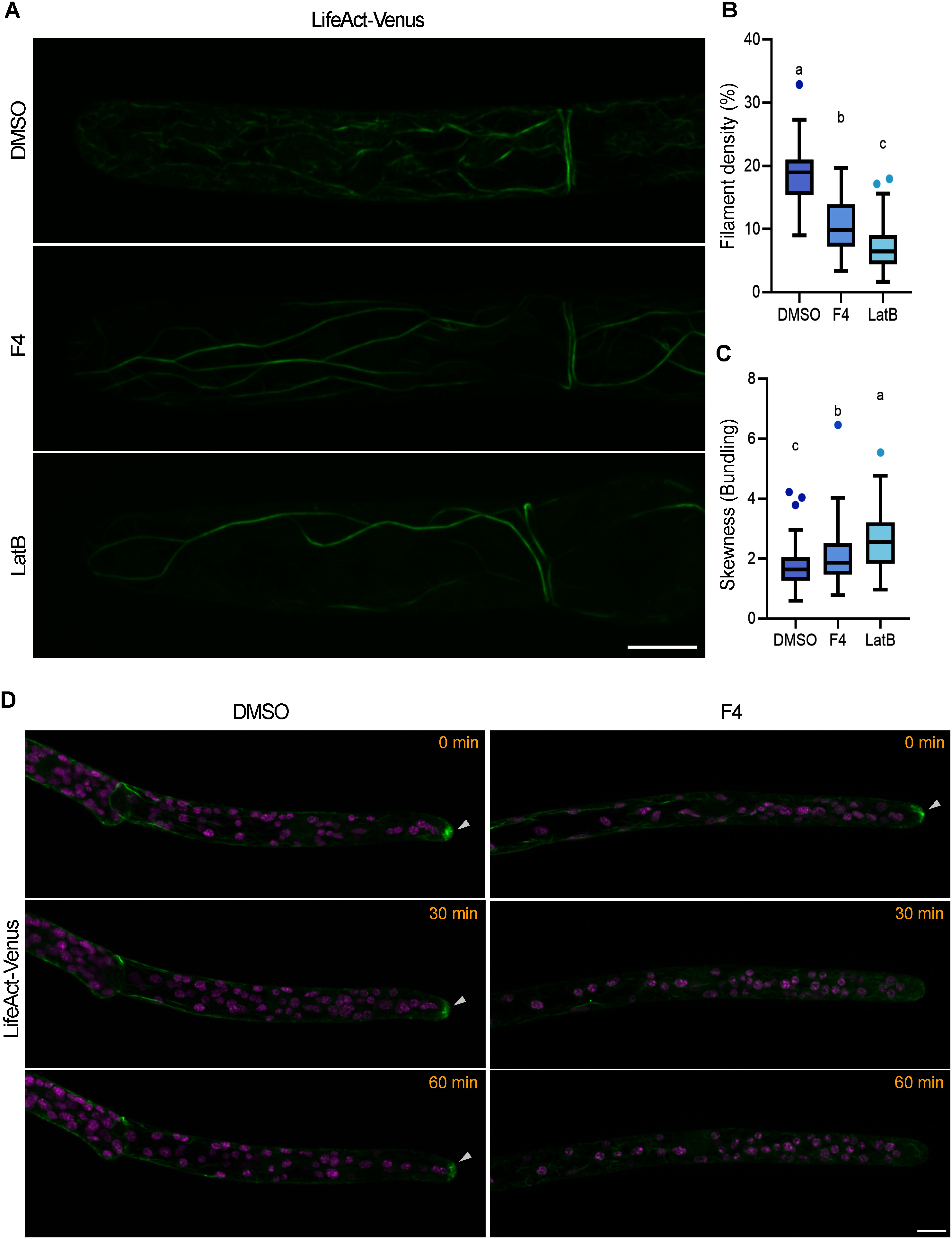
Figure 3. Reagent F4 induces actin depolymerization and loss of actin foci. A. Confocal images of 6-day LifeAct-Venus transgenic protonema grown in BCD media in glass bottom dishes, treated with 0.1% DMSO as control, 100 µM Reagent F4, and 1 µM latrunculin B, after 2 h of treatment. Scale bar, 10 µm. B. Quantification of filament density (denoted in percentage) of actin filaments in LifeAct-Venus transgenic protonema, treated with 0.1% DMSO (*n*=88) as control, 100 µM Reagent F4 (*n*=88) and 1 µM latrunculin B (*n*=101), after 2 h of treatment. Different letters indicate statistically significant differences (one-way ANOVA and Tukey’s multiple comparison test *p*<0.01). C. Quantification of skewness of fluorescence intensity denoting bundling of actin filaments in LifeAct-Venus transgenic protonema, treated with 0.1% DMSO (*n*=88) as control, 100 µM Reagent F4 (*n*=88) and 1 µM latrunculin B (*n*=101), after 2 h of treatment. Different letters indicate statistically significant differences (one-way ANOVA and Tukey’s multiple comparison test *p*<0.05). D. Confocal images of 6-day LifeAct-Venus transgenic protonema grown in BCD media in glass bottom dishes, treated with 0.1% DMSO (*n*=16) as control and 100 µM Reagent F4 (*n*=19). Timepoint 0 min denotes before treatment with the indicated compounds. The green channel represents LifeAct-Venus, and the magenta channel represents chlorophyll. White arrowheads indicate actin foci. Scale bar, 10 µm.

Due to the defective cell elongation and tip growth phenotypes upon Reagent F4 treatment, we sought to explore whether alterations in the organization of cytoskeletal elements were the underlying mechanism driving these physiological changes in cell shape and size. The cytoskeletal elements were observed by utilizing LifeAct-Venus transgenic line for actin filaments and GFP-Tubulin transgenic line for microtubules. Our observations indicated that even an acute exposure of approximately two hours to Reagent F4 resulted in a significant reduction in the overall density of actin filaments within the protonemata (Figure 3A, B). Notably, F4 treatment, like latrunculin B (LatB), an actin polymerization inhibitor, appeared to promote actin bundling, resulting in persistent, bright, long cable-like filaments (Figure 3A, C). This suggests that the treatment induces actin network depolymerization. Interestingly, acute exposure to Reagent F4 did not induce noticeable disorganization of the microtubule network, unlike the effects observed with oryzalin treatment (Supplementary Figure S2). This suggests that the primary impact of Reagent F4 on polarized cell expansion is likely mediated through its specific effects on the actin cytoskeleton, rather than through disruption of the microtubule network. Previous studies have also reported defective actin filament organization leading to stunted cell elongation and perturbed tip growth in moss (Augustine et al. 2011; Harries et al. 2005; Vidali et al. 2007), indicating the importance of actin filaments in tip growth of *P. patens* protonemata.

At the tip of protonemal apical cells, dense F-actin filaments accumulate into spot-like structures (Finka et al. 2007), termed as foci. These foci predict the sites of cell expansion (Wu and Bezanilla 2018) and help establish polarized cell expansion. Under the effect of Reagent F4, the actin foci in most of the apical cells were lost over the course of an hour (*n*=17/19, Figure 3D). These observations suggest that Reagent F4 impedes tip growth by perturbing the organization of the actin cytoskeleton.

### Reagent F4 affects ROP polar localization

In the protonema of *P. patens*, ROP displays a compact apical gradient in the plasma membrane of the apical cells (Cheng et al. 2020; Yi and Goshima 2020). Our observations above noted a loss of tip growth, prompting us to investigate how Reagent F4 impacts this important polarity regulator, ROP.

**Figure 4:**
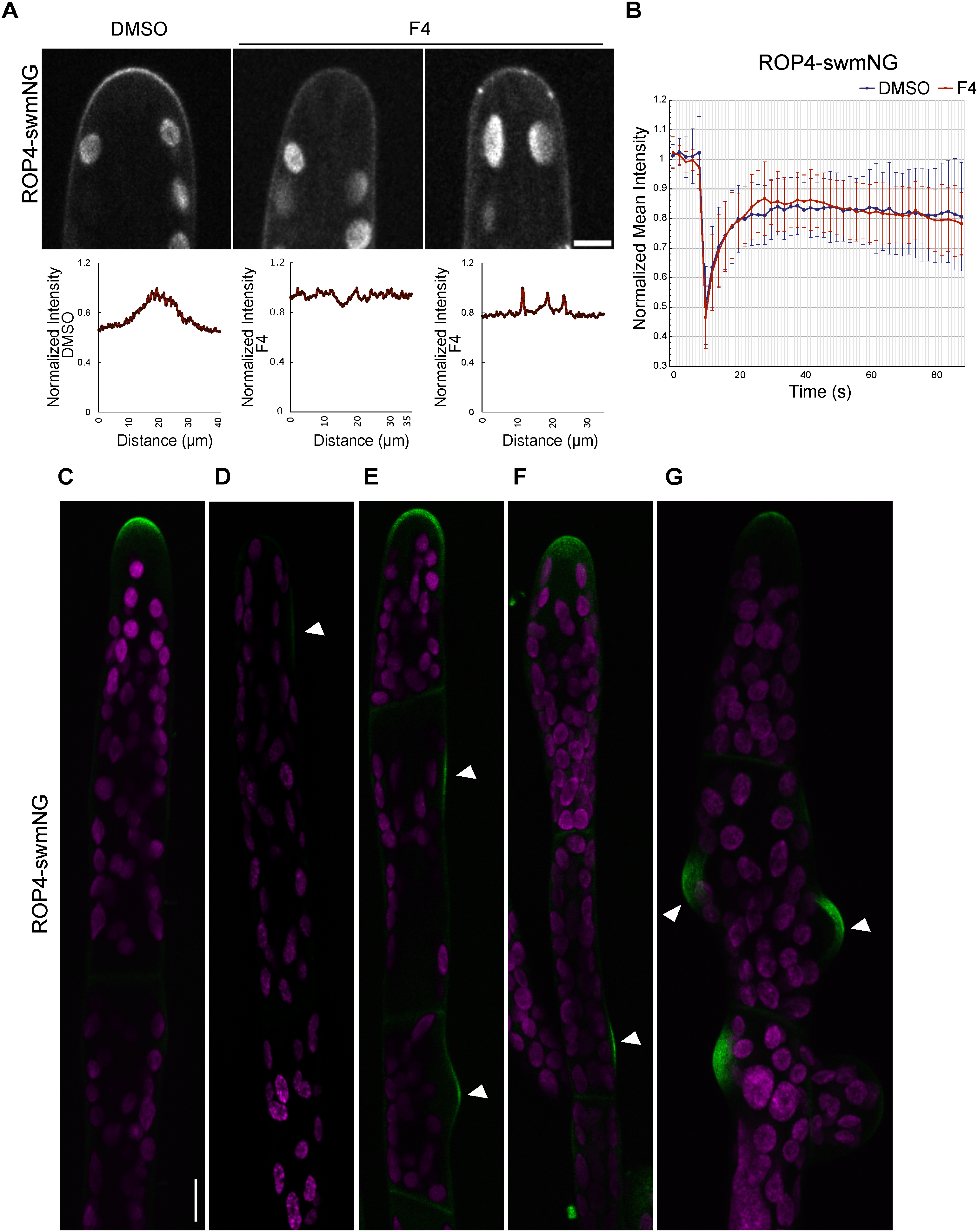
Figure 4. Short-term treatment with Reagent F4 causes loss of apical ROP gradient in apical cells. A. Representative confocal images of 6-day-old ROP4-swmNG transgenic protonema grown in BCDATG media in glass bottom dishes, treated with 0.1% DMSO (*n*=24) as control and 100 µM Reagent F4 (*n*=40), after 30 min of treatment and signal intensity quantification of protonemal apical cell after treatment with the indicated compounds. Graphs were generated by measuring the signal intensity along a manually drawn line tracing the plasma membrane of the cell. Scale bar, 5 µm. B. FRAP analysis of ROP4-swmNG, treated with 0.1% DMSO (*n*=16) as control and 100 µM Reagent F4 (*n*=19), after 30 min of treatment. The normalized mean fluorescence intensity of ROP4-swmNG was measured in the photobleached region of interest (ROI) and plotted over time. Each line is the average normalized intensity of all cells with the indicated treatment. Error bars represent standard deviation. C. Long-term exposure to Reagent F4 reveals aberrant ROP localization in *P. patens*. Confocal images of 7-day-old ROP4-swmNG transgenic protonema grown in BCDATG media in glass-bottom dishes, treated with 0.1% DMSO as a control, observed after 24 h of treatment. Scale bar, 10 µm. D.-G. Long-term exposure to Reagent F4 reveals aberrant ROP localization in P. patens. Shown are confocal images of 7-day-old ROP4-swmNG protonemal cells treated with 100 µM Reagent F4 for 24 hours, under the same growth conditions as (C). White arrowheads indicate ectopic localization of ROP in apical and subapical cells.

In actively growing tip cells, the apical gradient of ROP is maintained. However, acute treatment with Reagent F4 resulted in the loss of this gradient in approximately 73% of the observed apical cells (*n*=29/40) as opposed to the mock samples that did not exhibit a complete loss of ROP gradient (Figure 4A). Notably, this disappearance occurs within 30 min of Reagent F4 application, resembling the previously reported effects of LatB on the ROP gradient (Figure 3A, Cheng et al. 2020). Considering the underlying cause for the loss of the ROP apical gradient, we sought to determine whether Reagent F4 influences ROP dynamics at the cell apex. To investigate this, we employed fluorescence recovery after photobleaching (FRAP) to assess the ROP signal’s mobility following 30 min of Reagent F4 treatment. Our results showed that Reagent F4 did not significantly alter the recovery of the ROP signal; both control and treated samples exhibited similar recovery times, approximately 40 s (Figure 4B, Supplementary Movie S1).

ROP is also essential for branch formation, as it accumulates at the sites of side branch initiation along the lateral wall. Notably, ROP predicts future side branch formation by localizing to the apicolateral portion of subapical cells several hours before the protrusions occur (Cheng et al. 2020). To further investigate whether F4 treatment affects ROP localization along the lateral walls, we examined ROP distribution in protonemata after longer exposure to F4. While prolonged exposure to F4 diminished ROP localization at the tips of some apical cells, we observed ectopic ROP localization in several cells (Figure 4D–G). For instance, after extended treatment of F4 for 24 h, ROP accumulated on the lateral sides of apical cells (Figure 4D), and aberrantly at the medial side of both subapical and third cells (Figure 4E). Additionally, basal ROP accumulation was noted in subapical cells (Figure 4F), indicating potential side branch formation from unusual middle or basal regions. Consistently, some cells displayed abnormal side branch protrusions from the medial region of subapical cells (Figure 4G), where ROP was correctly localized at the tips of these protrusions. These findings suggest that F4 treatment disrupts ROP localization along the lateral wall, resulting in abnormal side branch formation at atypical positions. Hence, our results indicate that while Reagent F4 does not alter ROP dynamics, it may lead to the loss of ROP signaling due to specific spatial changes in ROP localization.

### Reagent F4′s mechanism of action through transcriptomics

**Figure 5:**
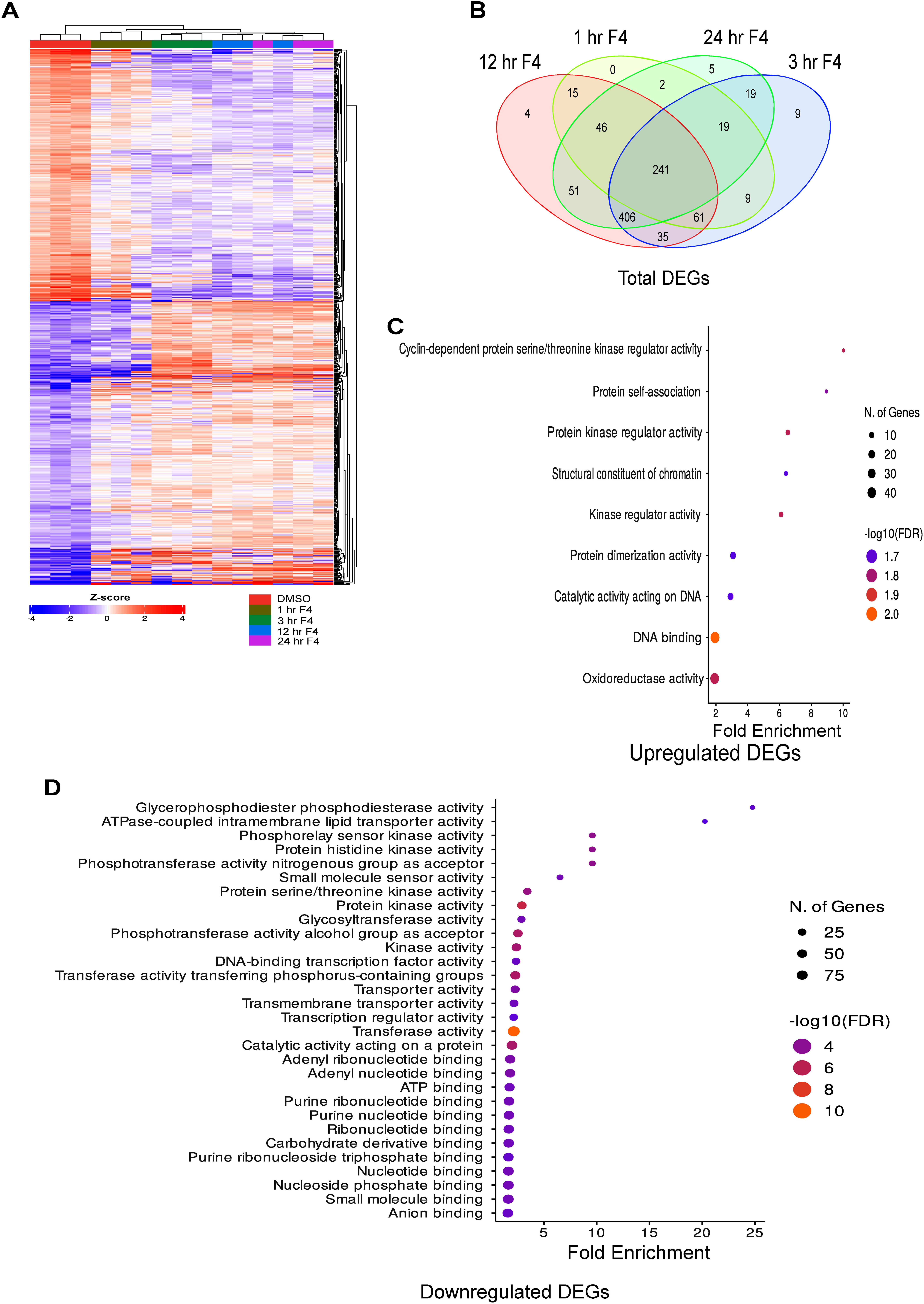
Figure 5. Transcriptional changes in response to short-term and long-term Reagent F4 treatment. A. Hierarchical clustering of normalized counts of the most differentially expressed genes (DEGs) of Reagent F4 treated samples at different time points, compared to 0.1% DMSO as mock. Clustering of samples was done, along with centering of the genes. Max Z-score=4. B. Venn diagram of the total differentially expressed genes, across different treatments and time points. C. A dot plot that depicts enriched GO terms: Molecular functions of the upregulated DEGs across all time points. The GO terms were sorted by fold enrichment. The size of the dots denotes the number of genes, and the color corresponds to the log10 (FDR) value, where orange is the high value and purple is the low value. D. A dot plot that depicts enriched GO terms: Molecular functions of the downregulated DEGs across all time points. The GO terms were sorted by fold enrichment. The size of the dots denotes the number of genes, and the color corresponds to the log10 (FDR) value, where orange is the high value and purple is the low value.

Since physiological changes were observed under both acute and sustained, long-term influence of Reagent F4, we wanted to identify the underlying factors corresponding to the physiological changes brought about by exposure to Reagent F4. To better understand the transcriptional landscape upon Reagent F4 application, RNA sequencing analyses were performed with WT protonemata treated with 100 µM Reagent F4 at 4 different time points (1, 3, 12, and 24 h), with 0.1% DMSO as a mock control. Differential gene expression analyses were carried out with DESeq2 and filtered for genes with adjusted *p*-*value*<0.05 and absolute log2FC value>0.58. The reproducibility of the experimental data was confirmed by principal component analysis, showing distinct expression patterns of Reagent F4 treated samples, compared to the mock samples (Supplementary Figure S3A). The similarities between the individual biological replicates demonstrate the high reproducibility of the measurements. Hierarchical clustering of normalized counts of the most differentially expressed genes (DEGs) revealed a significant shift in the transcriptome after just one hour of acute F4 treatment (Figure 5A). Additionally, replicates clustered together at 12 and 24 h, indicating similar expression patterns at these time points. Compared to mock samples, samples treated with Reagent F4 generated 924 DEGs across all time points. We identified the highest number of DEGs at 12 h after F4 treatment, and 241 genes showed common changes across all F4 chemical treatments (Figure 5B, Supplementary Table S1).

Among the upregulated DEGs, cyclin-dependent kinase activity (GO:0016538, GO:0019207, GO:0019887) -related GO terms were significantly enriched in the upregulated DEGs (Figure 5C). Though we did not observe any significant transcriptional changes of PpCDKA1, PpCDKA2, PpCDKB1 or PpCDKB2 (Bao et al. 2022), we found that genes involved in G1-to-S phase transition (CYCD) as well as G2-to-M phase transition (CYCA/CYCB) (Inzé and Veylder 2006) exhibited increased transcript levels (Supplementary Table S2). This indicates that Reagent F4 might influence cell cycle-related genes in *P. patens* protonemata, although any phenotypes substantiating this hypothesis have not been observed yet.

Gene Ontology (GO) enrichment analysis (Supplementary Table S2) of the downregulated DEGs showed that the GO Molecular Functions, glycerophosphodiester phosphodiesterase activity (GO:0008889), intramembrane lipid transporter activity (GO:0140303, GO:0140326), sensory histidine kinase activity (GO:0000155, GO:0004673, GO:0016775), and serine/threonine kinase activity (GO:0004674, GO:0004672) -related GO terms were highly enriched (adjusted FDR *p*≤0.05) (Figure 5D). Consistent with these GO terms, the GO biological processes related to cellular responses to internal and external stimuli (Supplementary Figure S3C) were enriched, as were the GO Cellular Components associated with the plasma membrane (GO:0005886, GO:0031226, GO:0005887) and cell periphery (GO:0071944) (Supplementary Figure S3D). These GO terms suggest that Reagent F4 might suppress polarized cell expansion by influencing lipid asymmetry and transport at the plasma membrane. Of all the downregulated DEGs, the genes involved in ATPase-coupled intramembrane lipid transport activity known as plant P4 ATPases/Aminophospholipid ATPases (ALAs) or lipid flippases are particularly interesting. Lipid flippases catalyze the translocation of lipids from the extracellular to the cytosolic side, maintaining transbilayer lipid asymmetry, which plays a role in establishing and maintaining cell polarity (Zhang et al. 2020), tip growth in pollen tubes (Poulsen et al. 2008; Yang et al. 2022; Zhou et al. 2020), and cell expansion (Davis et al. 2020). In our Reagent F4-treated dataset, P4 ATPase levels steadily declined over time (Supplementary Figure S4A).

## Discussion

Cell polarity refers to the asymmetric organization of cellular components essential for growth and development. Plant cells establish cell polarity for directional tip growth or cell shape determination (e.g., root hairs, pollen tubes or pavement cell) (Hepler et al. 2001; Settleman 2005). Polarity is driven by the localized distribution of signaling molecules, proteins, lipids, cytoskeletal elements, and vesicle trafficking pathways, enabling plants to adapt to environmental cues and ensure proper organ formation and tissue differentiation. In this study, we established an unbiased chemical screening system using *P. patens* protonemata and demonstrated the role of small molecules in regulating tip growth. We identified Reagent F4, a small molecule that perturbs tip growth by inducing loss of the apical gradient of ROP in protonema apical cells and depolymerization of the actin cytoskeleton, thereby affecting cell expansion, therefore, tip growth of protonemata. Transcriptomic analyses of the effect of Reagent F4 revealed a significant impact on the modulation of genes involved in lipid transport. From these findings, we speculate that Reagent F4 influences tip growth. Reagent F4 induces loss of apical ROP gradient in apical cells and ectopic localization in the lateral membranes of protonemal cells. Although we examined the transcriptomic regulation by F4 on RNA-seq analysis, we did not find any significant changes in the transcript levels of the 4 *P. patens* ROPs (Eklund et al. 2010) and the ROP signaling-related genes like ROPGAP, RENGAP, ROPGDI, RIC (Bascom et al. 2019; Eklund et al. 2010) except ROPGEF2 (Supplementary Figure S4B) where we observed an upregulation of ROPGEF2 across the time course dataset. ROPGEFs convert ROP from its inactive to active form (GDP-to-GTP) and drive polarized cell expansion by forming clusters with ROP (Ruan et al. 2023). Silencing of ROPGEFs in *P. patens* leads to small plants (Bascom et al. 2019). Further investigations are required to confirm the effect of upregulation of ROPGEF2. ROP has been implicated in angiosperms as being essential for accurate actin organization and assembly (Fu et al. 2001, 2002). In our study, Reagent F4 treatment disrupted actin filament organization. The polar accumulation of ROP relies on its prenylation motif and interactions with anionic phospholipids at the plasma membrane (Saavedra et al. 2011; Yi and Goshima 2020). Therefore, it is possible that the mislocalization of ROP, triggered by perturbation of ROP-membrane interactions upon Reagent F4 application, contributed to the loss of polarized actin foci and subsequent actin filament depolymerization.

It is unclear whether Reagent F4 interacts or binds with ROP or actin directly, but studies in yeast have shown that appropriate polarized localization of the yeast ortholog of ROP, Cdc42, is facilitated by transbilayer lipid flipping (Saito et al. 2007). Lipid asymmetry at the plasma membrane, mediated by the lipid flippase complex, enables fast Cdc42 recycling (Das et al. 2012). Plant P4 ATPases, also known as aminophospholipid ATPases (ALAs) or lipid flippases, transport lipids from the extracellular to the cytosolic side of the membrane. *Arabidopsis* contains 12 P4 ATPases, with varying degrees of characterization. ALA3-deficient mutants exhibit mislocalization of polarized PIN1 protein from the basal to the apical membrane in epidermal cells (Zhang et al. 2020) and defective pollen tube growth (Zhou et al. 2020). Loss of ALA3 function also disrupts polar localization of apical phosphatidylserine (PS), indicating its critical role in establishing and maintaining apical PS distribution in pollen tubes. Furthermore, *Arabidopsis* ROP6 has been shown to bind to phosphatidylinositol-3,5-bisphosphate (PtdIns(3,5)P2), phosphatidylinositol-4,5-bisphosphate (PtdIns(4,5)P2) and other anionic phospholipids like PS (Zhou et al. 2020), suggesting that ROP can directly interact with a variety of lipid substrates. These reports, along with our transcriptomics data where lipid flippases-related GO terms were highly enriched in the downregulated DEGs, suggest that lipid asymmetry mediated by flippases in *P. patens* might regulate interaction with small GTPases like ROP and affect its localization by changing the lipid distribution throughout the bilayer. Defective lipid asymmetry also leads to inefficient recruitment of pollen-specific receptor kinases in Arabidopsis pollen tubes (Yang et al. 2022), which activate ROPGEF in pollen tubes, leading to ROP activation. Additionally, lipid asymmetry is required for the activation of the Arp2/3 complex that promotes actin filament assembly (Bezanilla et al. 2015; Saarikangas et al. 2010). Identifying the lipid environment of the plasma membrane under the influence of Reagent F4 could provide deeper insights into the substrate specificity of *P. patens* ROP. This may also reveal upstream factors involved in ROP recruitment to the membrane, ultimately regulating polarized tip growth in *P. patens*.

Several reports have utilized small molecules to investigate plant development (Drakakaki et al. 2011; Kimata et al. 2023; Sakai et al. 2017) and we report a novel chemical, Reagent F4, that affects polarized cell expansion by causing defects in cell elongation, actin polymerization, and proper ROP localization maintenance in *P. patens*. Identification of Reagent F4 target proteins provides valuable knowledge in clarifying the mechanisms involved in tip growth and polarized cell expansion in *P. patens* and in plants, in general, as ROP and actin-based tip growth regulation appear to be well conserved (Orr et al. 2020).

## Data Availability

The RNA-seq dataset presented in this study has been deposited with links to BioProject accession number PRJNA1226400 in the NCBI BioProject database (https://www.ncbi.nlm.nih.gov/bioproject/ (Accessed Feb 21, 2025)). According to the American Chemical Society database (SciFinder), Reagent F4 is currently available from companies in Canada, the US, Hong Kong, and France. Reagent F4 is commercially available from vendors, AMBINTER (France) (https://ambinter.com/molecule/6333422) and A2B (USA) (https://www.a2bchem.com/853891-69-7.html).

## Abbreviations

DEG: differentially expressed gene
DMSO: dimethyl sulfoxide
FDR: false discovery rate
FRAP: fluorescence recovery after photobleaching
GFP: green fluorescent protein
GO: Gene Ontology
RNA-seq: RNA sequencing
ROI: region of interest
SAR: structure-activity relationship
TPM: transcripts per million
WT: wild type

## Description of supplementary files

Supplementary Figure S1. Reagent F4 affects cell elongation by reducing growth rate.

Supplementary Figure S2. Reagent F4 does not affect microtubule organization post-acute treatment in *P. patens*.

Supplementary Figure S3. Bulk RNA-seq of short-term and long-term Reagent F4 treated *P. patens*.

Supplementary Figure S4. Expression level changes of genes-of-interest upon Reagent F4 treatment.

Supplementary Table S1. Differential gene expression analysis of *P. patens* treated with Reagent F4.

Supplementary Table S2. GO term enrichment analysis of upregulated and downregulated DEGs of *P. patens* treated with Reagent F4.

Supplementary Movie S1. FRAP analysis of ROP4-swmNG under the effect of Reagent F4. Pale yellow circles indicate the ROI for photobleaching.
