# Supplementary material for: Novel small molecules disrupting polarized cell expansion and development in the moss, Physcomitrium patens
Source: Plant Biotechnol (Tokyo). 2025 Jun 25;42(2):131–43. doi: 10.5511/plantbiotechnology.25.0209a (PMC12235423; doi:10.5511/plantbiotechnology.25.0209a)
Supplement: Supplementary Data [file plantbiotechnology-42-2-25.0209a-s001.pdf]

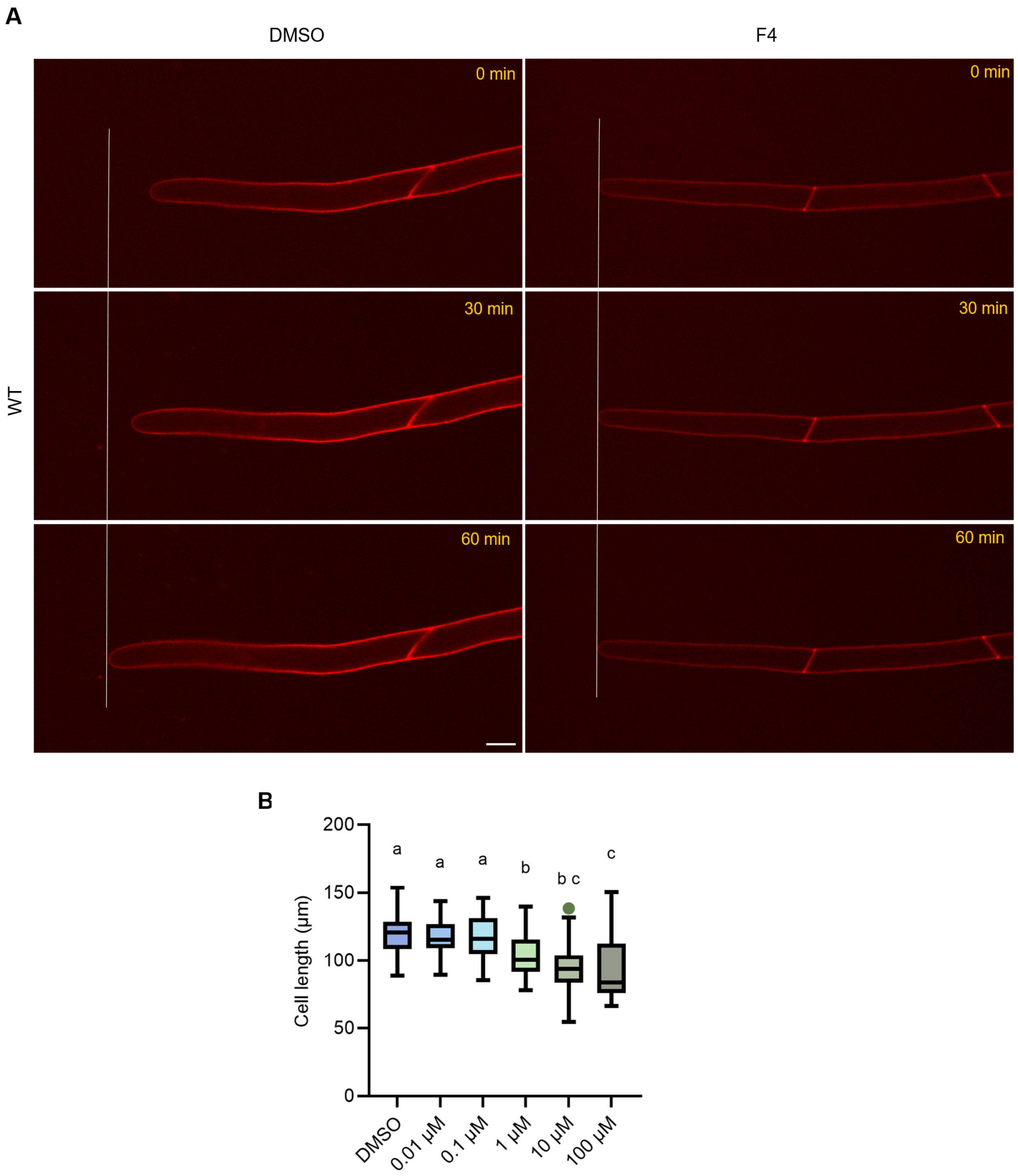

Supplementary Figure S1. Reagent F4 affects cell elongation by reducing growth rate.

A. Confocal images of 5-day WT protonema grown in BCD media in glass bottom dishes, treated with 0.1% DMSO (n = 9) as control and 100 µM Reagent F4 (n = 12). Timelapse was started 20 minutes after treatment with the indicated compounds. Timepoint 0 minutes

indicates the beginning of the observation. Samples were stained with PI to indicate cell wall and septa. The apical cell continued to grow in the mock samples but not in samples treated with Reagent F4. White lines indicate the same X-axis position. Scale bar, 20 µm.

B. Quantification of 2nd subapical cell (3rd cell from the apical cell) length after treatment with 0.1% DMSO and different concentrations of Reagent F4, after one day of treatment (n = 63, 65, 44, 50, 37, 52). Different letters indicate statistically significant differences (one-way ANOVA and Tukey's multiple comparison test $p < 0.01$).

A

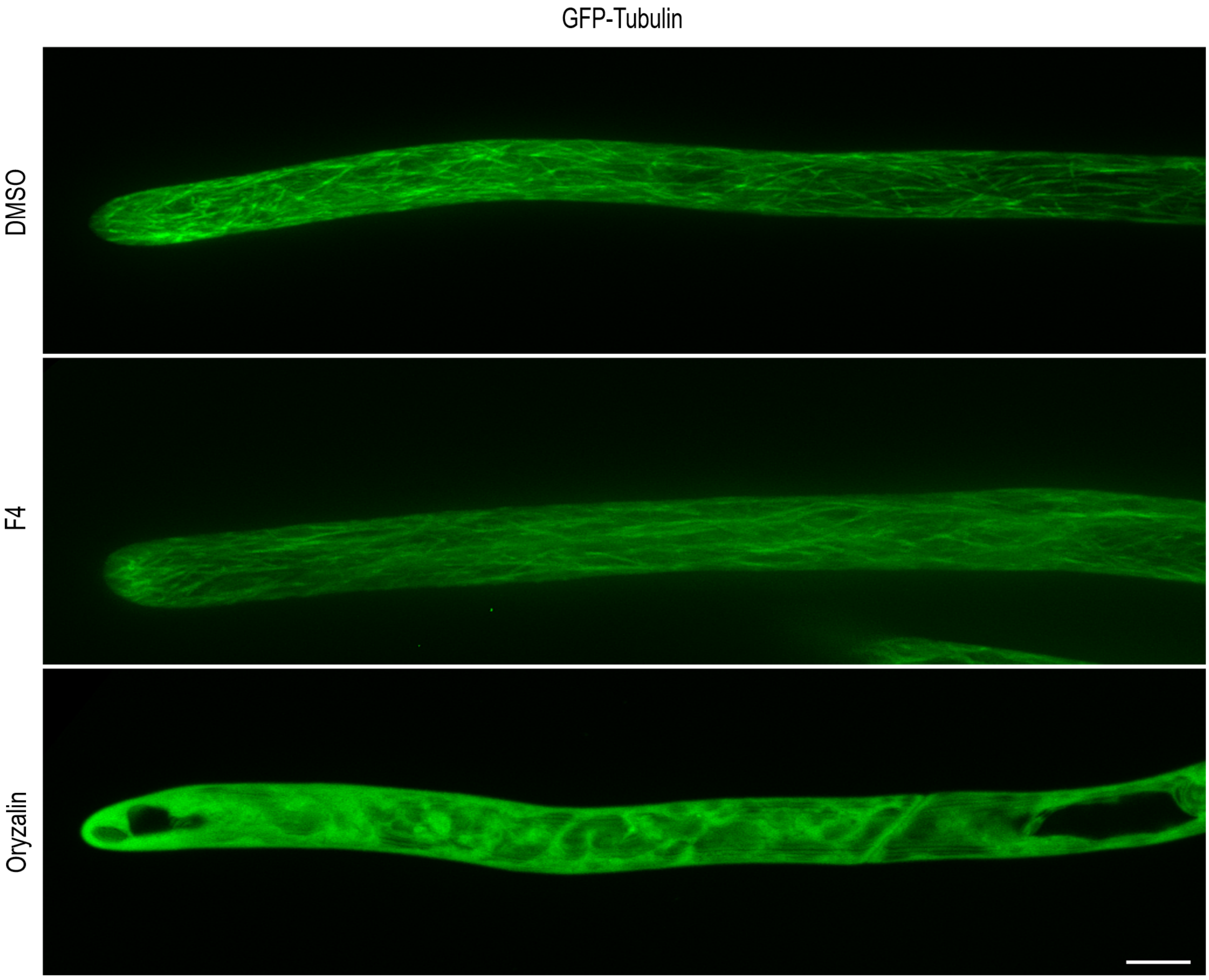

Supplementary Figure S2. Reagent F4 does not affect microtubule organization post-acute treatment in *P. patens.*

Confocal images of 6-day GFP-Tubulin transgenic protonema grown in BCD media in glass bottom dishes, treated with 0.1% DMSO as control (n = 20), 100 µM Reagent F4 (n = 22) and 10 µM Oryzalin (n = 13), after 2 hours of treatment. Scale bar, 10 µm.

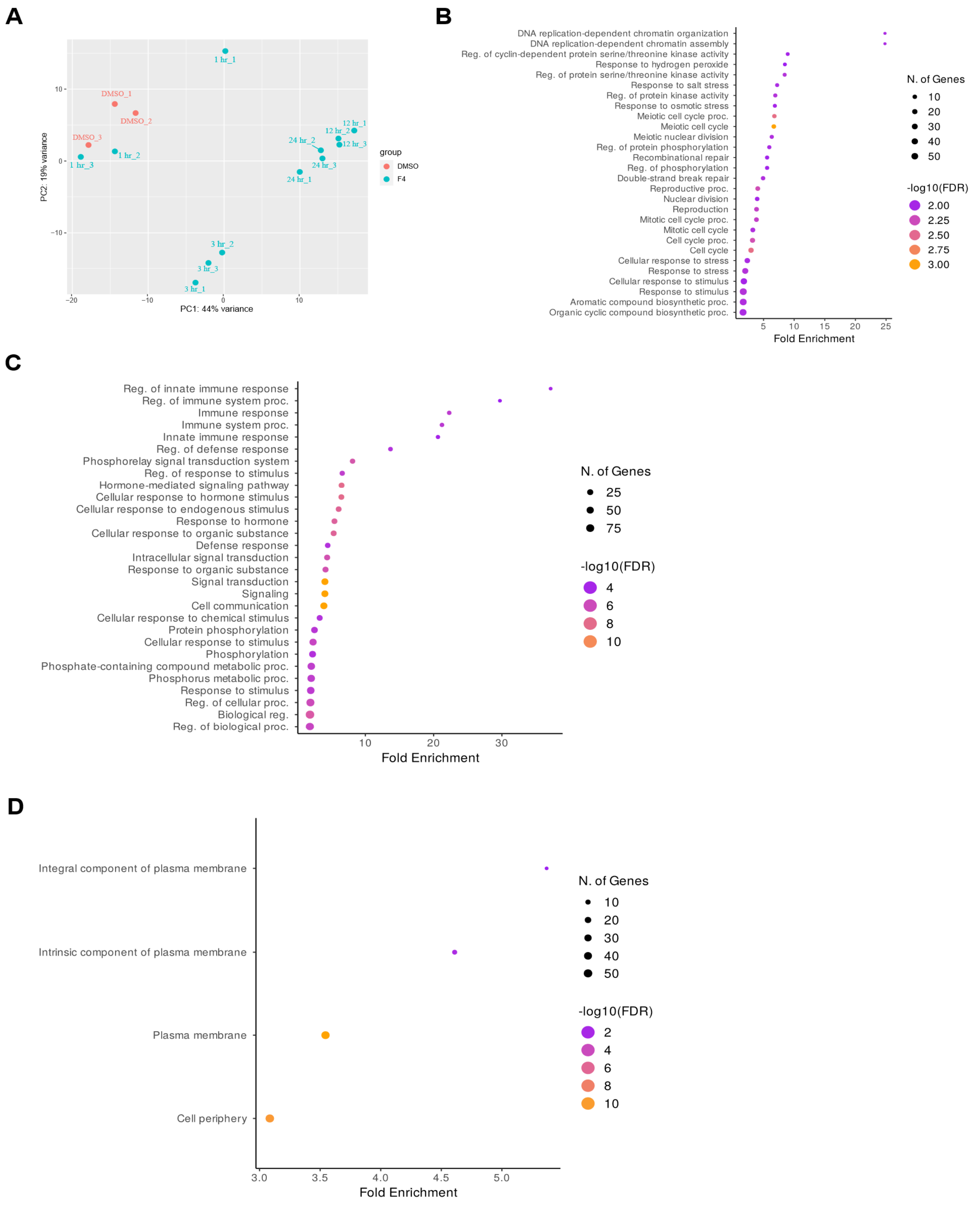

Supplementary Figure S3. Bulk RNA-seq of short-term and long-term Reagent F4 treated *P. patens.*

A. Principal component analysis of all the replicates of samples at different time points.
B. A dot plot that depicts enriched GO terms: Biological Processes of the upregulated DEGs across all time points. The GO terms were sorted by fold enrichment. The size of the dots denotes the number of genes, and the color corresponds to the log10 (FDR) value, where orange is the high value and purple is the low value.
C. A dot plot that depicts enriched GO terms: Biological Processes of the downregulated DEGs across all time points. The GO terms were sorted by fold enrichment. The size of the dots denotes the number of genes, and the color corresponds to the log10 (FDR) value, where orange is the high value and purple is the low value.
D. A dot plot that depicts enriched GO terms: Cellular Components of the downregulated DEGs across all time points. The GO terms were sorted by fold enrichment. The size of the dots denotes the number of genes, and the color corresponds to the log10 (FDR) value, where orange is the high value and purple is the low value.

A

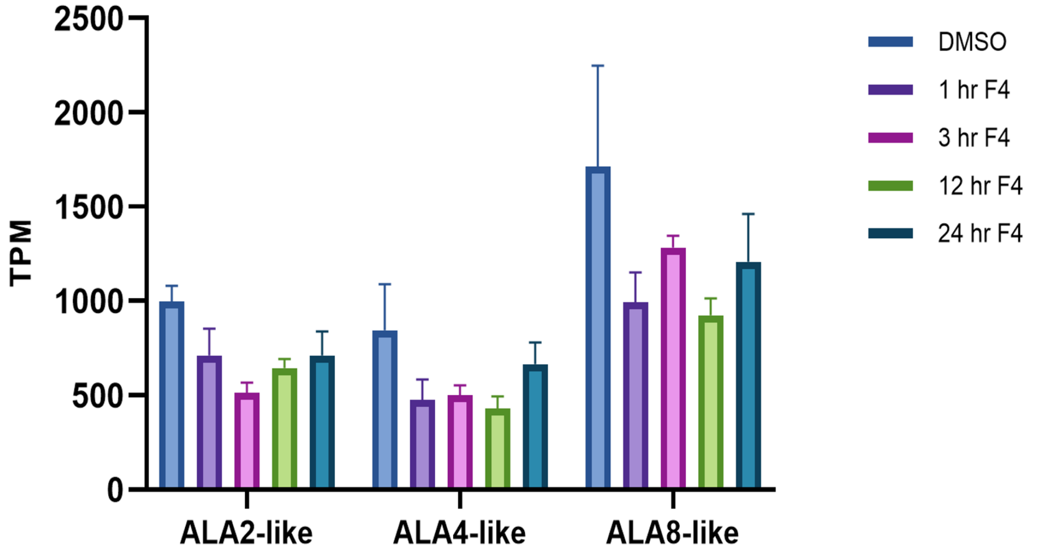

B

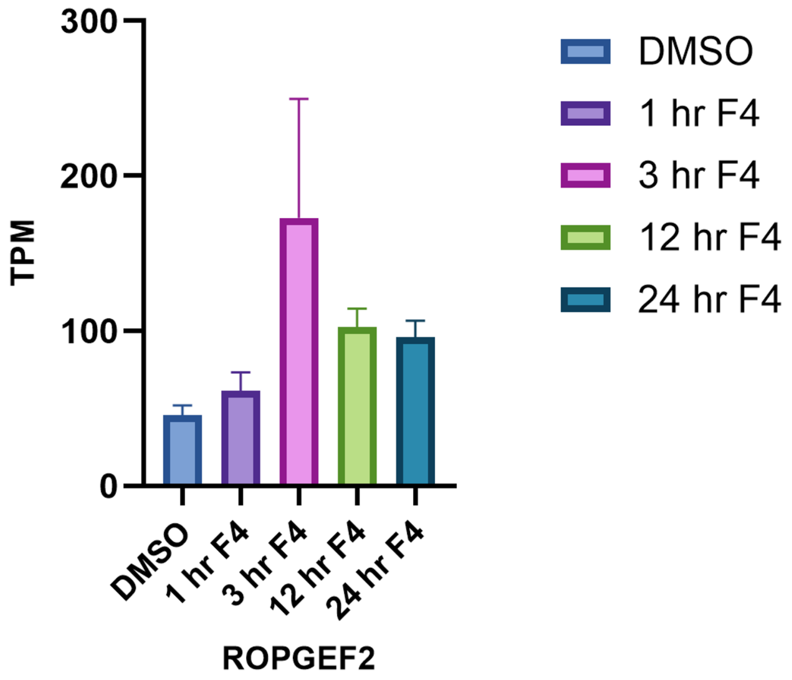

Supplementary Figure S4. Expression level changes of genes-of-interest upon Reagent F4 treatment.

A. Gene expression profiles of the downregulated lipid flippases in *P. patens* in Reagent F4 treated samples at different time points. The X-axis represents the time points, and the y-axis represents the normalized TPM values with standard deviation (SD) error bars.

B. Gene expression profiles of the upregulated ROPGEF2 in *P. patens* in Reagent F4 treated samples at different time points. The X-axis represents the time points, and the y-axis represents the normalized TPM values with standard deviation (SD) error bars.
